# optCluster: An R Package for Determining the Optimal Clustering Algorithm

**DOI:** 10.6026/97320630013101

**Published:** 2017-03-31

**Authors:** Michael Sekula, Somnath Datta, Susmita Datta

**Affiliations:** 1 Department of Bioinformatics and Biostatistics, University of Louisville, Louisville, Kentucky, 40202, USA;; 2 Department of Biostatistics, University of Florida, Gainesville, Florida, 32611, USA;

**Keywords:** Clustering, Validation, Gene Expression, RNA-Seq

## Abstract

There exist numerous programs and packages that perform validation for a given clustering solution; however, clustering algorithms fare differently as judged by different validation measures. If more than one performance measure is used to evaluate multiple clustering partitions, an optimal result is often difficult to determine by visual inspection alone. This paper introduces optCluster, an R package that uses a single function to simultaneously compare numerous clustering partitions (created by different algorithms and/or numbers of clusters) and obtain a “best” option for a given dataset. The method of weighted rank aggregation is utilized by this package to objectively aggregate various performance measure scores, thereby taking away the guesswork that often follows a visual inspection of cluster results. The optCluster package contains biological validation measures as well as clustering algorithms developed specifically for RNA sequencing data, making it a useful tool for clustering genomic data.

**Availability:**

This package is available for free through the Comprehensive R Archive Network (CRAN) at http://cran.rproject.org/web/packages/optCluster/

## Background

Cluster analysis is playing an increasingly important role with many of the new advancements in the field of bioinformatics. Researchers often attempt to expose underlying structures inside large datasets in hopes that molecular profiles are organized into biologically meaningful clusters. For example, genes with similar biological functions would ideally be grouped into the same cluster. When “little” or “no” information is known about the data, clustering algorithms are necessary for assigning similar observations together into the same group. Different algorithms naturally create different clustering partitions, so choosing the “best” partition can be a potentially daunting endeavor. Since clustering is an unsupervised learning task, there is no correct answer to what the “best” partition for a dataset really is. Therefore, researchers turn to validation measures as a method of comparing multiple clustering results. These validation measures evaluate clusters based on specific performance criteria such as compactness, separation, or biological homogeneity. An inherent problem with cluster validation is that a clustering algorithm may perform well for one index but perform poorly for another. In fact, it is quite common for a different choice of validation measure to result in a different optimal clustering solution. The detailed article by [1] examines the potential issues associated with cluster validation in the field of bioinformatics and suggests using multiple validation indices to compare different partitions. Unfortunately, choosing the “best” clustering option based on a visual inspection of multiple validation results can be problematic since the results typically consist of multiple “best” options. Hence, an algorithmic method that can provide a unique solution to this problem would be useful.

In this paper, we present an R [2] package called optCluster to help researchers establish an optimal or “best” solution to cluster analysis. Using a single function call, the user is able to evaluate various combinations of clustering algorithms and numbers of clusters with multiple validation measures, and determine the most ideal clustering partition for a particular dataset through an aggregation of ranked validation measure lists.

## Methodology

The core of the optCluster package is weighted rank aggregation, which was proposed by [3] as a technique to evaluate a set of validation measure results. Clustering partitions, created by different algorithms and/or numbers of clusters, can be sorted into a ranked list, Vi, based on their performances for a specific validation index. When using more than one validation measure in a cluster analysis, a set of ranked lists is created. The idea behind rank aggregation is to combine these lists together and generate an optimally ranked list that is as close as possible to the entire collection of original lists. In this context, rank aggregation is essentially an optimization problem. Suppose there are m number of ranked lists (V1, … , Vm) such that each Vi is associated with a corresponding importance weight wi. An objective function for a proposed list L can be defined as

$\Phi (L)=\overset{m}{\underset{\mathrm{i=1}}{\sum }}w_{i}\times \mathrm{dist}(L¸V_{i}{)}^{´}$

where dist(L,Vi) represents a distance function. The ultimate goal is to use an aggregation algorithm to minimize this objective function and find some list L* that has the smallest total distance between itself and all of the Vi’s. In the simplest sense, all of the ranked lists can be given the same value of importance weight; however, a researcher may choose to weigh lists differently for more control over the aggregation process. For example, one might choose to give validation indices with more relevance heavier weights while down-weighting indices measuring similar characteristics.

### Software input

The optCluster package extends the popular validation package clValid [4] by producing an optimal clustering solution based on rank aggregation of multiple validation scores and including clustering algorithm options appropriate for genomic count data. The main function, optCluster, has a simple interface that only requires the user to input a minimum of four initial arguments. These arguments are: ‘obj’ = dataset, ‘nClust’ = number of clusters, ‘clMethods’ = clustering algorithm names, and ‘validation’ = validation measure types. The user must provide a matrix or data frame for the ‘obj’ argument (where the samples to be clustered are the rows of the data) and input a range of numbers of clusters (or a specific number of clusters) for ‘nClust’. For the other two arguments, the optCluster package provides a variety of options for the user to select from. These options are displayed in table 1.

The ten clustering algorithms offered by clValid for continuous data are available for the user to input into the ‘clMethods’ argument, as well as six model-based clustering approaches for RNA sequencing (RNA-Seq) read count data (or any other count data). The expectation maximization (EM) algorithm and two of its variations, the deterministic annealing (DA) algorithm and the simulated annealing (SA) algorithm, were proposed by [5] for clustering RNA-Seq count data based on a mixture of either Poisson or negative binomial distributions. Combining each related algorithm with each discrete distribution results in the six available options for clustering count data in this package.

There are nine possible validation measures offered by optCluster that are categorized into three distinct types for the ‘validation’ argument input: “internal”, “stability”, and “biological”. Internal validation measures examine the statistical properties of clusters while using only information from the dataset and the created clustering partition. Stability validation measures are specialized internal measures that evaluate whether the cluster assignments remain stable with respect to small perturbations to the dataset. Biological validation measures are external measures that assess the performance of an algorithm to produce clusters of biologically similar genes in an analysis of gene expression data such as microarray or RNA-Seq data.

For weighted rank aggregation, this package provides the weighted Spearman’s footrule and the weighted Kendall’s tau as possible distance measures, and the cross-entropy Monte Carlo algorithm and the genetic algorithm as options for aggregation algorithms. Details on the use of these measures and algorithms in rank aggregation can be found in [3,6]. As default, optCluster assumes equal weights on each validation measure list while using the weighted Spearman’s footrule distance and the cross-entropy Monte Carlo algorithm. The user may change these options with the arguments of ‘importance’, ‘distance’, and ‘rankMethod’, respectively. In addition to the arguments listed above, the optCluster function has a variety of other arguments to allow the analysis to be fine-tuned according to the user’s preferences. A complete list of these arguments is included in the reference manual of the package.

### Software output

An S4 object of class “optCluster” is generated as the output from the optCluster function. This object contains detailed information on the created clustering partitions, calculated validation measure scores, and optimized rank aggregation results. In addition to obtaining the “best” clustering partition, this package includes several methods developed specifically for accessing the information contained within an “optCluster” object. For viewing the results from cluster validation, the valScores method outputs the calculated scores for each validation measure of interest and the optimalScores method displays the top performing clustering algorithm and number of clusters for each measure. The ordered lists used in rank aggregation consisting of either clustering partitions or validation scores are extracted with the methodRanks and scoreRanks methods, respectively. Additionally, clustering results for a specific algorithm are obtained with the clusterResults method.

### Comparison to similar packages

There are only a handful of packages currently available in R that offer some form of simultaneous optimization of cluster analysis with respect to multiple validation measures. Two packages, MOCCA [7] and NbClust [8], both find an appropriate number of clusters for a dataset, but only for a limited number of clustering algorithms. MOCCA provides a multi-objective optimization of validation indices to determine an optimal number of clusters based on three different clustering algorithms and four validation measures. NbClust finds the ideal number of clusters for either the K-means algorithm or a hierarchical clustering algorithm by analyzing up to thirty validity indices. A third package, COMMUNAL [9], offers one function that evaluates up to fourteen clustering algorithms across up to eighteen performance measures and a second function that combines the results of selected clustering algorithms to create a clustering partition for a given number of clusters. This package, however, does not generate the optimal number of clusters for the user. Instead, it relies on the user to determine the “best” number based on a visualization of performance measure results.

The optCluster package is unique compared to these three packages because it provides a solution (both clustering algorithm and number of clusters) using a single function. In addition, algorithms for clustering count data and validation measures for evaluating clusters based on biological gene functionality are included in this package but not in the others.

A cluster analysis was performed on a subset of RNA-Seq count data from [10] to compare the results obtained by optCluster to the results provided by the three similar packages. After normalizing the data (which is available in the optCluster package) with respect to library size, the appropriate function in each package was run using a range of two to four clusters and all possible validation measures and clustering algorithms suitable for continuous data. Hierarchical clustering using the Unweighted Pair Group Method with Arithmetic Mean (UPGMA) and three clusters was chosen by optCluster as the optimal clustering algorithm and number of clusters for this normalized dataset. The visualization produced by COMMUNAL agreed with this result as it suggested that three clusters was ideal, and the NbClust analysis, using either UPGMA or K-means, also selected three clusters as the most appropriate number. MOCCA was the only package that provided a different result, as it determined four clusters to be the “best”. With the continuous data, the optimal number of clusters determined by optCluster was consistent with two out of the three similar packages. Interestingly, when optCluster was run on the count data, two clusters was determined to be the ideal number with the “best” clustering algorithm being the DA algorithm based on the negative binomial distribution.

### Caveats and future development

The optCluster package is suitable for a variety of different cluster analyses. As new clustering and validation methodology is developed for genomic data, future developments may include adding more options for validation measures and clustering algorithms.

## Figures and Tables

**Table 1:** Clustering algorithm and validation measure options offered by the optCluster package. Clustering algorithms are selected individually and divided into two categories: continuous data and count data. Validation measures are selected in groups and divided into three classifications: internal, stability, and biological.

Clustering algorithms	
Continuous data	Hierarchical
	Agnes
	Diana
	K-means
	Pam
	Clara
	Fanny
	Model-based
	SOM
	SOTA
Count data	EM negative binomial
	DA negative binomial
	SA negative binomial
	EM Poisson
	DA Poisson
	SA Poisson
Validation measures	
Internal	Connectivity
	Dunn index
	Silhouette width
Stability	Average proportion of non-overlap
	Average distance
	Average distance between means
	Figure of merit
Biological	Biological homogeneity index
	Biological stability index
